# Acute Hepatitis Induced by Intravesical BCG Therapy: A Rare but Serious Complication

**DOI:** 10.1155/2021/4574879

**Published:** 2021-06-24

**Authors:** Hakima Abid, Mouna Figuigui, Sidi Adil Ibrahimi, Mohamed El Abkari, Mohamed Mzyiene, Soufiane Ennaciri, Ouima Justin Ziba, Mohamed Jamal El Fassi, Layla Tahiri Elousrouti, Laila Chbani

**Affiliations:** ^1^Department of Hepato-Gastroenterology, University Hospital Hassan II-Fez, Laboratory of Human Pathology, Biomedicine and Environment, Fes, Morocco; ^2^Faculty of Medicine and Pharmacy of Fez, Sidi Mohamed Ben Abdellah University Fez-Morocco, Fes, Morocco; ^3^Urology Department-University Hospital Hassan II-Fez, Fes, Morocco; ^4^Pathological Anatomy Department, University Hospital Hassan II of Fez, Fes, Morocco

## Abstract

Bacillus Calmette and Guérin (BCG), widely used as a vaccination to prevent tuberculosis, is also used as immunotherapy, by intrabladder instillation, to treat superficial bladder cancers and prevent recurrence. Complications following instillation of BCG are most often localized reactions, such as cystitis or hematuria. They can more rarely be generalized and potentially severe such as hepatitis, pneumopathies, aortitis, and localization to hematopoietic tissue. We have reported the observation of a 47-year-old patient followed up for a bladder tumor operated for transurethral resection of the bladder, then having benefited from an instillation of BCG therapy complicated by occurrence a week later of an acute hepatitis. The diagnostic time was 2 days, and the outcome was favorable with corticosteroid therapy.

## 1. Introduction

Bacillus Calmette and Guérin (BCG) is a living strain of *Mycobacterium bovis* of attenuated virulence. Its main uses are the vaccination for the prevention of tuberculosis and the immunotherapy of superficial urothelial cancers. Intravesical instillation is, therefore, indicated as a first-line treatment as a complementary curative treatment to endoscopic resection and for the prevention of recurrence. BCG therapy can be complicated by systemic attacks such as pneumonia, hepatitis, aortitis, bone marrow failure, spondylodiscitis [1].

## 2. Observation

This is a 47-year-old patient who has been a chronic tobacco user for 20 years and gave up 4 years ago. He already had a surgery for left varicocele in 2005. The history of the disease dates back to October 2020 when the patient had undergone a transurethral resection of the bladder, including the anatomopathological study of the shavings of resection returned in favor of a pTa G2 tumor. The decision was to put the patient on BCG therapy that had started 4 months after surgery. The patient had suddenly presented one day after the first instillation of the BCG a cholestatic jaundice associated with an influenza syndrome made up of asthenia and myalgia with pain in the right hypochondrium all evolving in a context of feeling feverish, chills, and asthenia. The examination has shown a conscious, afebrile, jaundiced patient with hypogastric tenderness and right hypochondrium without hepatomegaly.

**Figure 1:**
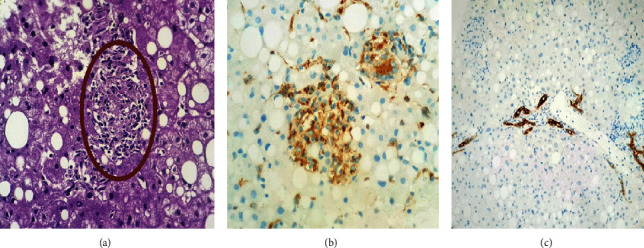
Histological results of the liver biopsy puncture of our patient (laboratory of pathological anatomy, CHU Hassan II of Fez). (a) Epitheloid granuloma without giant cells and without caseous necrosis (HPS ∗ 200). (b) Anti-CD68 antibody highlighted epitheloid granulomas on immunohistochemistry. (c) Anti-CK19 antibody showed portal cholangiolar proliferation on immunohistochemistry.

Biology has revealed a disturbed liver function test with ASAT: 245 U/L (6 times the upper limit of normal (ULN)), ALAT: 487 (12 ∗ LSN), GGT: 611 U/L (8.7 LSN), PAL: 237 U/L (1.4 ∗ LSN), total bilirubin: 64, and direct bilirubin at 41. CRP was 7.5 mg/L. Thrombocytopenia was noted at 68000 elements/mm^3^. Hemoglobin was correct at 16 g/dl as well as leukocytes (5140/mm^3^), PT (100%), and renal function (creatinine = 8 g/l, urea = 0.29). The liver ultrasound and abdominal CT scan were normal except a fatty liver. The serologies for hepatitis A, B, C, HIV, CMV, and EBV were negative. Protein electrophoresis and immunoglobulin weight assay were normal. There were no antimitochondria or antitissue antibodies or antineutrophilic cytoplasm (ANCA). Blood cultures remained sterile. Thus, a hepatic biopsy was performed, and the anatomopathological analysis revealed granulomatous steatohepatitis without giant cells or caseous necrosis, classified S3A2F1, made up of an inflammatory lobular infiltrate and granulomas made of epithelioid and histiocytic cells; a special staining by Masson's trichrome was performed showing portal fibrosis without septa emissions and sinusoidal fibrosis, and special reticulin staining showed retention of the reticulin pattern. Immunohistochemical complement was performed. CD 68 highlighted granulomas described above, and CK 19 demonstrated portal cholangiolar proliferation (Figure 1).

In the absence of obvious infectious or inflammatory disease, the diagnosis of granulomatous hepatitis secondary to BCG therapy was retained. The patient was put on injectable corticosteroid therapy at a dose of 1 mg/kg/day for 15 days and then degression. The clinical course was marked by the improvement of asthenia and jaundice. Assessment at the first month after the initiation of treatment has found that the patient was in good condition with a biological improvement. The treatment continued during 3 months with a remarkable clinical improvement and a total normalization of the hepatic biological assessment (Table 1).

## 3. Discussion

Immunotherapy by intravesical instillation of Bacillus Calmette–Guérin (BCG) is currently the most effective treatment for noninfiltrating bladder tumors. Furthermore, it is contraindicated if there is active tuberculosis or a history of local or systemic complications due to this therapy. As sometimes this is an unrecognized pathology that can occur long after instillations, it is also important to inform the patient about the possible complications to avoid any delay in treatment.

Three pathophysiological mechanisms have been described to explain the AEs of BCG therapy:

- Infectious: linked to the proliferation and dissemination by the hematogenous route of BCG. The innate, nonspecific immune reaction against BCG has been responsible for a local inflammatory reaction [2].
- Immunoallergic: related to a type IV hypersensitivity reaction of the Gell and Coombs classification. This hypersensitivity has been explained by the production of IL10 which directs the immune reaction towards a Th2 response.
- Autoimmune: linked, on the one hand, to cross-immune reactions between BCG antigens and “self-proteins” and, on the other hand, to a suppression of the regulatory immune mechanisms considered to be protective against autoimmunity [3].

The main side effects are local, such as cystitis, hematuria, prostatitis, and epididymitis. General effects are more rarely described [4]. The diagnosis of complications of BCG therapy is sometimes difficult, especially since they can occur long time afterward, up to several years after the last instillation. It is based on a set of clinical, biological, radiological, microbiological, and pathological factors and will most often be a diagnosis of elimination [5]. Any fever occurring after an instillation of BCG should raise suspicion of the diagnosis of BCGitis. It will be necessary to look for an associated organ damage by a complete clinical examination, a biological assessment (particularly renal and hepatic assessment), and a radiological assessment including at least a chest X-ray. An inflammatory syndrome is often associated. It is important to rule out active tuberculosis, another infectious process, metastases or other causes of granulomatous lesions depending on the type of organ affected [6]. The time between instillations and systemic manifestations is very variable, from a few days to several years. This may be explained by the prolonged persistence of BCG in urothelial tissue, at least 16 months after instillation [7]. Symptoms that occurred early would preferentially concern the lungs, liver, and joints, while late damage would be rather related to arterial aneurysms and spondylodiscitis [8]. As far as our patient is concerned, severe liver damage occurred two days after the first instillation while it is noted after 5 weeks in the case of Özbakkaloğlu et al. [9].

Pathological examination for a gigantocellular granuloma, most often without caseous necrosis, appears to be of major diagnostic interest. In a series of 22 patients, it enabled the diagnosis in 71% of cases, and in a review of the literature on 183 biopsied patients, an inflammatory granuloma was found in 86% of cases [7]. In our observation, we have found granulomatous hepatitis without caseous necrosis. The classical causes of this type of attack have been ruled out: autoimmune hepatitis and primary biliary cirrhosis, chronic viral hepatitis, and lymphoma.

In the presence of caseous necrosis, the specific curative treatment is that of infection with *M. bovis*, a germ that is constantly resistant to pyrazinamide, and must, therefore, combine rifampicin, isoniazid, and ethambutol as a first-line treatment. The duration of treatment is not codified as some cases are treated for 6 to 12 months [9–11]. In our patient, corticosteroid therapy was used in a good progress. It is frequently indicated in disseminated disorders, but its use is not based on studies with a sufficient level of proof to determine its usefulness, the doses to be used, or the duration of treatment. Nevertheless, its use is recommended by learned societies in the event of a systemic adverse effect, at a dosage of 0.5 to 1 mg/kg/day for 10 to 15 days maximum [10]. It should be remembered that compliance with the contraindications of BCG therapy remains major. A period of at least 15 days must be observed between tumor resection and instillation of BCG [12], which postpones the instillation in the event of traumatic catheterization, in the event of an infectious process with fever and/or positive ECBU, or in the event of local signs such as dysuria, hematuria, and urination burns.

Drug prophylaxis has been evaluated twice. Administration of isoniazid (INH) 300 mg daily for 3 days starting on the day of instillation did not show any effect on the occurrence of local and systemic side effects. Administration of ofloxacin at H6 and H18 after the first postinstillation urination showed a significant reduction in the incidence of systemic side effects (by 21.5%) and a reduction in the number of discontinuation of instillations following the instillation. Occurrence of side effects: this practice seems interesting but has not been studied any further. It is not recommended in current practice but should be considered. The reduction in the consumption of antituberculosis drugs in the treatment of complications from BCG therapy in the ofloxacin versus placebo group makes it a very interesting practice which deserves special attention [12].

## 4. Conclusions

Our observation illustrates a rare but serious and curable complication of intravesical BCG therapy, especially visceral diffusion in the liver. There is no specific preventive treatment to avoid such a complication, and it, therefore, appears essential to know this complication and to make an early diagnosis to limit any delay in treatment.

## Figures and Tables

**Table 1:** Results and evolution of our patient's labs.

	Two days after BCG therapy	Second day of steroids therapy	One month after steroids therapy
ASAT	6 ULN	6 ULN	1ULN
ALAT	11, 8 ULN	10, 8 ULN	1, 9 ULN
GGT	243	611	127
PAL	225	237	103
Total bilirubin	64	25	25
Direct bilirubin	41	22	19
PT	100	100	80
Platelets	68000	419000	425000
